# Gut microbiota restoration through fecal microbiota transplantation: a new atopic dermatitis therapy

**DOI:** 10.1038/s12276-021-00627-6

**Published:** 2021-05-20

**Authors:** Jong-Hwa Kim, Kiyoung Kim, Wonyong Kim

**Affiliations:** Department of Microbiology, Chung-Ang University College of Medicine, Seoul, South Korea

**Keywords:** Experimental models of disease, Biological therapy

## Abstract

The pathogenesis of atopic dermatitis (AD) involves complex factors, including gut microbiota and immune modulation, which remain poorly understood. The aim of this study was to restore gut microbiota via fecal microbiota transplantation (FMT) to ameliorate AD in mice. FMT was performed using stool from donor mice. The gut microbiota was characterized via 16S rRNA sequencing and analyzed using Quantitative Insights into Microbial Ecology 2 with the DADA2 plugin. Gut metabolite levels were determined by measuring fecal short-chain fatty acid (SCFA) contents. AD-induced allergic responses were evaluated by analyzing blood parameters (IgE levels and eosinophil percentage, eosinophil count, basophil percentage, and monocyte percentage), the levels of Th1 and Th2 cytokines, dermatitis score, and the number of mast cells in the ileum and skin tissues. Calprotectin level was measured to assess gut inflammation after FMT. FMT resulted in the restoration of gut microbiota to the donor state and increases in the levels of SCFAs as gut metabolites. In addition, FMT restored the Th1/Th2 balance, modulated Tregs through gut microbiota, and reduced IgE levels and the numbers of mast cells, eosinophils, and basophils. FMT is associated with restoration of gut microbiota and immunologic balance (Th1/Th2) along with suppression of AD-induced allergic responses and is thus a potential new therapy for AD.

## Eczema: Fecal transplant reduces inflammation in mice

Fecal transplants that restore a healthy gut microbiome could help quell the immune processes responsible for eczema, an itchy skin condition also known as atopic dermatitis. Wonyong Kim and colleagues from Chung-Ang University College of Medicine in Seoul, South Korea, transplanted fecal samples from healthy mice to mice with experimentally induced eczema. After the transplant, the mice showed signs of a healthier gut ecosystem, as evidenced by the presence of certain favorable bacterial metabolites. The treatment also restored a more favorable immune balance and lowered the blood levels of certain inflammatory cells and signaling molecules associated with allergic responses. The mice exhibited fewer signs of skin irritation. The findings suggest that human testing of the intervention is warranted and some small trials have already begun.

## Introduction

Atopic dermatitis (AD) is a chronic inflammatory skin disease known to affect ~15–30% of children and 10% of adults^1^. The pathogenesis of AD is a complex combination of the immune response, impaired barrier function, and microbial factors, but it remains poorly understood^2^. Many studies have attributed changes in skin microbiota to immune modulation due to disturbances in epidermal barrier function^3^. However, there has been a recent focus on gut microbiota in association with immune modulation as a factor of interest in AD^4^.

The gut microbiota is composed of 100 trillion resident bacteria and plays an important role in gut homeostasis and immunity^5^. Restoration of gut homeostasis is considered a potential target for therapeutic strategies, since dysbiosis of gut microbiota is thought to be influenced by various diseases^6,7^. Many studies have discussed the relationships between gut microbiota and diseases such as AD, obesity, diabetes, immunological diseases, and neurological diseases^8–10^. Previous studies on AD have reported the effects of probiotic treatment on patients with AD through gut microbiota diversity manipulation or reduction, which were not always beneficial^3,11–13^. Moreover, the effective dosage and therapeutic potential of probiotics are unknown, as probiotics might be inhibited by the gastrointestinal tract, including the stomach^14^.

Fecal microbiota transplantation (FMT), a novel method for re-establishing gut microbiota^3^, allows long-lasting alteration of the recipient’s microbiome, whereas treatment with probiotics results in temporary colonization^15,16^. FMT has been successfully applied for some disorders, such as *Clostridium difficile* infection, inflammatory bowel disease, and diabetes, but it has not been applied for AD^17–19^. FMT may be more effective than probiotics for the treatment of AD. Therefore, the potential therapeutic effect of FMT was investigated by gut microbiota manipulation, immune system modulation, and fecal metabolite analysis using an AD mouse model.

## Material and methods

### Ethics and animals

Five-week-old female BALB/c mice (*n* = 60; antibiotic selection: *n* = 30; FMT experiment: *n* = 30) were purchased from the Central Lab Animal Incorporation (Seoul, South Korea) and adapted for 1 week. All mice were housed in a cage with free access to food and water. They were maintained under a 12-h light/dark cycle at 24 ± 2 °C and 55 ± 10% humidity. The animal experiment was approved by the Chung-Ang University Institutional Animal Care and Use Committee of the Laboratory Animal Research Center (IACUC No. 2017-00044) and conducted in accordance with the Korean Food and Drug Administration guidelines.

The mice were randomly divided into three groups (*n* = 10/group): (1) the donor group (non-ovalbumin [OVA]-sensitized + phosphate-buffered saline [PBS]), (2) before_FMT group (OVA-sensitized + PBS; AD induction), and (3) FMT_8w group (OVA-sensitized + fecal samples from donor mice). AD was induced according to the method described by Kim et al.^20^. To induce AD in mice by skin sensitization, the dorsal hair was shaved using electric clippers, and OVA grade V (Sigma-Aldrich, St. Louis, MO, USA) and alum (Sigma-Aldrich) in PBS were applied twice a week to the dorsal skin and administered by intraperitoneal injection on days 7, 21, 35, and 49 (Fig. S1).

The FMT group was subjected to FMT 1 week post-AD induction. All groups were sacrificed 8 weeks post-AD induction, and the dorsal skin, ileum, blood, and spleen were collected for further analysis. Fecal samples were collected 1 and 6 weeks after FMT for gut microbiome analysis.

### Antibiotic selection

Mice were administered one of five different antibiotics (*n* = 3/group), i.e., streptomycin, vancomycin, ampicillin, gentamycin, and the combination of streptomycin and vancomycin (Sigma-Aldrich), before FMT, which depleted the mouse gut microbiota. The antibiotics were suspended in sterile water at concentrations of 20 and 100 mg/ml, and 200 μl was administered by oral gavage. After oral antibiotic treatment, the mice were housed in a new cage with sterilized bedding. Fecal pellets were collected 24 and 48 h later, vortexed at maximum speed for 1 min, and centrifuged at 1000 × *g* for 5 min. The supernatants were then serially diluted and plated on brain heart infusion agar (BD Difco, Sparks, MD, USA), and the total number of colony-forming units (CFUs) was determined after incubation at 37 °C overnight.

### Preparation for FMT

FMT was performed as described previously^21,22^. Prior to FMT, fecal pellets were collected from four donor BALB/c mice (the donor group), pooled, and placed in 1 ml transfer buffer containing sterile filtered 0.05% cysteine HCl (Sigma-Aldrich) in Dulbecco’s PBS (Sigma-Aldrich) on ice. Fecal pellets were homogenized and centrifuged at 3000 × *g* for 3 min at 4 °C. The supernatant was collected and diluted with transfer buffer at a ratio of 1:3. The gut microbiota of mice with AD was depleted following antibiotic treatment prior to FMT. The mice were housed again in a cage with sterile bedding after antibiotic treatment. Recipient mice (*n* = 10 per group) were orally inoculated with diluted fecal suspension (200 μl) for 2 weeks (a total of six times). The donor and before_FMT mouse groups were only administered 200 μl transfer buffer.

### Gut microbiome analysis

Fecal samples were collected from each mouse, immediately transported on ice, and stored at −80 °C. DNA was extracted from the fecal samples using the FastDNA SPIN kit for bacterial DNA (MP Biomedicals, Santa Ana, CA, USA) according to the manufacturer’s instructions. The V3–V4 region of the 16S rRNA gene was amplified via polymerase chain reaction and sequenced using MiSeq-based high-throughput sequencing (Illumina, San Diego, CA, USA). After sequencing, raw FASTQ files were processed using Quantitative Insights into Microbial Ecology 2 (QIIME2; version 2020.08) software^23^. The sequences were filtered and trimmed to remove low-quality, short, and chimeric reads using the DADA2 plugin in QIIME2^24^. The quality reads were Q-25, and the read length was 300 bp. The data table was imported into R software (version 4.0.0; National Institutes of Health, Bethesda, MD, USA), and normalized operational taxonomic unit (OTU) levels were used to analyze the alpha diversity (observed species, Chao1, and Shannon indices) and bacterial taxa and to generate a heatmap. The analysis was performed using the vegan^25^ and phyloseq^26^ R packages and visualized using the ggplot2 package in RStudio (version 1.3.959; RStudio, Boston, MA, USA). Beta diversity was analyzed using unweighted UniFrac metrics and was visualized using principal coordinate analysis (PCoA) plots in QIIME2. Taxonomy classification was performed using the classifier module (gg-13-8-99-515-806-nb-classifier.qza) of the Greengenes database using the “qiime feature-classifier classify-sklearn” command. The sequences generated in this study were deposited in the National Center for Biotechnology Information-Sequence Read Archive (http://www.ncbi.nlm.nih.gov/sra) under the accession numbers SRR12825157–SRR12825118.

### Analysis of short-chain fatty acid (SCFA) contents in fecal samples

Gut metabolite (SCFA) contents in mouse fecal samples were examined using high-performance liquid chromatography (HPLC; Ultimate 3000; Thermo Dionex, Sunnyvale, CA, USA). SCFA samples were prepared by homogenization of fecal samples and centrifugation at 12,000 × *g* for 20 min at 4 °C. SCFAs (acetic acid, butyric acid, isobutyric acid, and propionic acid) were separated using an Aminex 87H column (300 × 10 mm; Bio-Rad, Hercules, CA, USA) with an isocratic mobile phase (0.01 N sulfuric acid; Fluka, La Jolla, CA, USA) set at a flow rate of 0.5 ml/min and then identified at a wavelength of 210 nm using an RI detector (ERC; RefractoMAX520, Tokyo, Japan).

#### Blood and serum cytokine analysis

Whole blood samples were collected via retro-orbital sinus puncture from the medical canthus of the eye in spray-dried ethylenediaminetetraacetic acid tubes (Green Cross Laboratories, Yongin, South Korea) for analysis of the eosinophil percentage, eosinophil count, basophil percentage, and monocyte percentage. Blood serum was obtained by coagulation for 1 h at 4 °C and centrifugation for 1 h at 5000 × *g* for analysis of IgE, cytokine (IL-4, IL-5, IL-10, IL-12, IL-13, IL-1β, TNF-α, and IFN-γ), and calprotectin levels. Cytokines and calprotectin levels were analyzed using enzyme-linked immunosorbent assay kits (R&D Systems, Minneapolis, MN, USA, and Cusabio Biotech, Wuhan, China) according to the manufacturers’ instructions. The absorbance at 450 nm was measured using a NanoQuant Plate^TM^ (Tecan, Männedorf, Switzerland).

#### Flow cytometry analysis

Spleens were harvested from sacrificed mice and homogenized using a cell strainer (SPL, Pocheon, South Korea). The cells were counted using trypan blue with a TC10 automated cell counter (Bio-Rad, Hercules, CA, USA), diluted to a density of 2.0 × 10^6^ cells/tube, and stained with a phycoerythrin-labeled mouse anti-CD 86 or anti-CD 274 antibody (BD Pharmingen, San Jose, CA, USA), on ice for 20 min. The cells were analyzed by acquiring data for at least 10,000 events with a flow cytometer (FACSCalibur; Becton Dickinson, Franklin Lakes, NJ, USA), and mean fluorescence intensity data were collected using FACSCalibur Cell Quest software (v. 6.0).

### Evaluation of dermatitis scores and histological analysis

The severity of dermatitis was evaluated via skin severity scores after 1 week (start of sensitization), after 8 weeks (completion of sensitization), and 8 weeks post-AD induction. Severity scores were calculated as the sum of individual scores assigned as follows according to the occurrence of dryness, erythema, and scratching behaviors within 15 min of skin sensitization: 0 (none), 1 (mild), 2 (moderate), and 3 (severe). The mice were sacrificed for histologic examination. The dorsal skin and ileum were harvested, fixed in 10% formalin in PBS, and embedded in paraffin. Tissues were stained with toluidine blue (TB) for evaluation of mast cells. The number of mast cells in the dorsal skin and ileum sections was determined after observation of five random fields and all fields, respectively, using a DM 4000B microscope (Leica Microsystem, Wetzlar, Germany) at ×400 magnification.

### Statistical analyses

Significant differences in the microbiome among groups were evaluated using the R package. Multiple comparisons of the microbiomes of different groups were made using the Kruskal–Wallis test and Dunn’s post hoc test in GraphPad Prism software (v. 8.0). Permutational multivariate analysis of variance (PERMANOVA) (pairwise) was performed and PCoA plots were generated using QIIME2. The data are presented as the means ± standard errors of the mean. Significant differences among groups in SCFA contents, blood parameters, cytokine levels, flow cytometry results, and histologic analyses (except microbiome analysis) were evaluated via an unpaired two-tailed *t*-test for two groups and one-way analysis of variance for multiple groups. Results with a *p* value < 0.05 were considered statistically significant.

## Results

### Selection of antibiotics for FMT

**Fig. 1:**
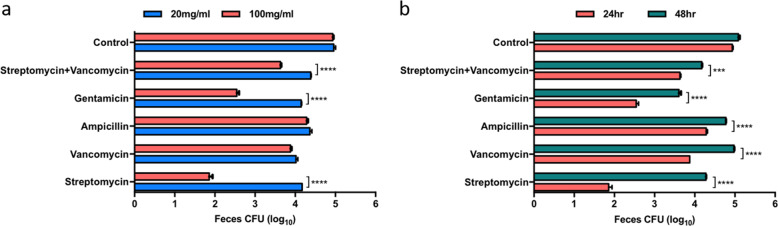
Selection of the optimal antibiotic for FMT. Optimal antibiotic treatment was determined by **a** the dosage and **b** the time of administration. Antibiotic efficiency is shown in the bar graph (***p < 0.0005; ****p < 0.0001). FMT fecal microbiota transplantation.

The mice were administered one of five antibiotics to deplete the existing gut microbiota in preparation for FMT. The concentration of each antibiotic and duration of pretreatment were determined by the number of CFUs in plated fecal samples. The effect of pretreatment with antibiotics at two concentrations, 20 and 100 mg/ml, was assessed (Fig. 1a). A significant decrease in richness was observed after 24 h of treatment with three antibiotics (gentamicin, streptomycin, and an antibiotic cocktail of streptomycin and vancomycin) at a concentration of 100 mg/ml compared with after 24 h of treatment with these antibiotics at a concentration of 20 mg/ml (*p* < 0.0001). After the optimal antibiotic concentration was determined, the number of CFUs was evaluated 24 and 48 h postantibiotic treatment (Fig. 1b). There were fewer CFUs after 24 h than after 48 h of treatment with all antibiotics tested (*p* < 0.001). Streptomycin (100 mg/ml) treatment resulted in the fewest CFUs (<2 log) after 24 h (*p* < 0.0001). Overall, the number of CFUs decreased after antibiotic treatment (*p* < 0.0001). These results demonstrate that oral administration of 100 mg/ml streptomycin for 24 h most effectively depletes gut microbiota before FMT.

### Change in the gut microbiota profile after FMT

Bacterial DNA was sequenced to investigate the effect of gut microbiota restoration following FMT. After filtration, the feature table had a total of 645 features and an 824,305 feature count at the specified sampling depth of 28,000 reads. There were an average of 34,346 sequences (ranging from 28,589 to 46,156) and 430 OTUs per sample.

**Fig. 2:**
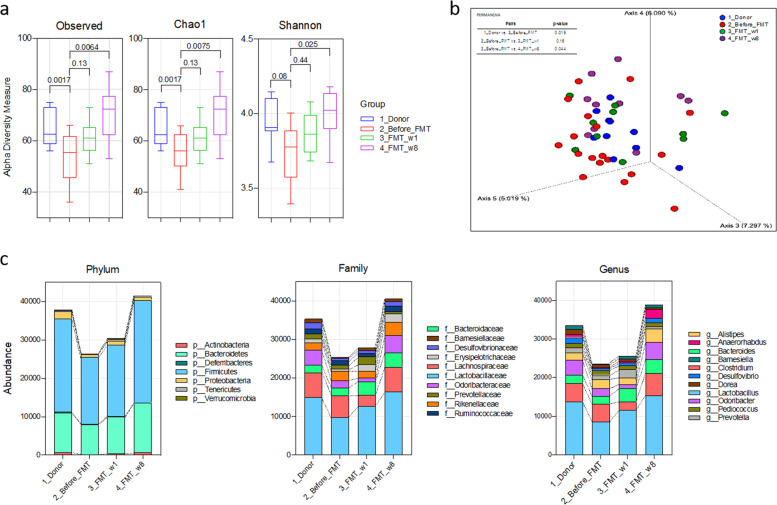
Effect of FMT on the gut microbiota. Gut biodiversity is displayed based on **a** alpha-diversity indices and **b** PCoA plots. **c** Bacterial abundance at the phylum, family, and genus levels is shown. FMT fecal microbiota transplantation, OTU operational taxonomic unit, PCoA principal component analysis.

The observed species, Chao1, and Shannon (alpha-diversity measure) indices revealed that there was greater microbial richness among groups in the FMT group than in the before_FMT group (Fig. 2a). All three indices increased significantly after FMT treatment (*p* < 0.0001), as in the donor group, and no difference in the indices was observed between the 1-week and 6-week post-FMT time points. Moreover, comparison of gut microbiota composition was performed using PCoA of beta diversity based on unweighted UniFrac distances. Gut microbial communities were clearly divided between the before_FMT and FMT (1 week and 8 weeks) groups. Variations observed among the gut microbial communities on the PCoA plot were supported by a significant difference between pairs of groups, i.e., the donor and before_FMT groups (PERMANOVA, *p* = 0.019, *F* = 1.49), the donor and FMT_1w groups (*p* = 0.077, *F* = 1.32), the donor and FMT_8w groups (*p* = 0.001, *F* = 2.52), the before_FMT and FMT_1w groups (*p* = 0.160, *F* = 1.23), the before_FMT and FMT_8w groups (*p* = 0.014, *F* = 1.48), and the FMT_1w and FMT_8w groups (*p* = 0.014, *F* = 1.72) (Fig. 2b).

Abundance changes in the gut microbiota at the taxonomic level were also evaluated. The dominant bacterial phyla were Actinobacteria, Bacteroidetes, Firmicutes, and Proteobacteria (Fig. 2c and Fig. S2a). Compared with the before_FMT group, the FMT_1w and FMT_8w groups showed a significantly higher abundance of Actinobacteria (*p* = 0.013 and 0.025, respectively), Bacteroidetes (*p* = 0.048 and 0.018), and Firmicutes (*p* = 0.007 and 0.019). The abundance of the phylum Proteobacteria was reduced in the donor group (*p* = 0.037), but it did not differ significantly between groups. FMT treatment resulted in an increased abundance of the families *Barnesiellaceae*, *Desulfovibrionaceae*, *Erysipelotrichaceae*, *Lactobacillaceae*, and *Odoribacteraceae* (Fig. 2c and Fig. S2b). In further detail, at the genus level, the abundance of *Alistipes, Bacteroides, Barnesiella, Lactobacillus*, and *Odoribacter* was increased in the FMT-treated groups compared with the before_FMT group (Fig. 2c and Fig. S2c). In addition, the families *Bacteroidaceae*, *Lactobacillaceae*, *Odoribacteraceae*, and *Rikenellaceae* were more abundant in the donor, FMT_1w, and FMT_8w groups than the before_FMT group (Fig. S3).

### Increase in SCFA levels after FMT

**Fig. 3:**
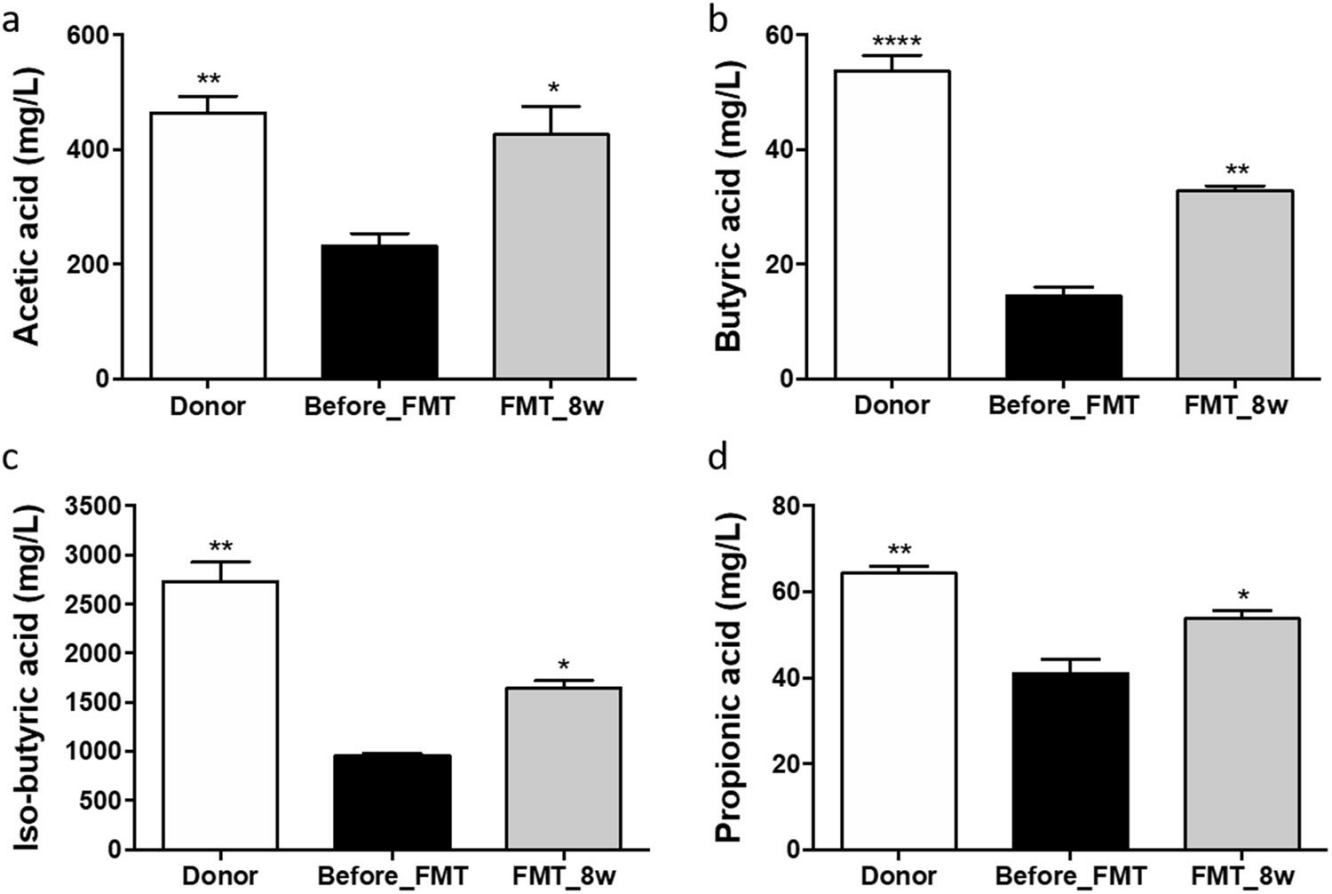
Influence of FMT on SCFA levels. The levels of SCFAs, including **a** acetic acid, **b** butyric acid, **c** isobutyric acid, and **d** propionic acid, as determined via HPLC, are shown (*p < 0.05; **p < 0.001; ****p < 0.0001). FMT fecal microbiota transplantation, HPLC high-performance liquid chromatography, SCFA short-chain fatty acids.

To evaluate the effect of FMT on gut metabolites, SCFA (acetic, butyric, isobutyric, and propionic acid) levels were examined via HPLC. There were differences in the concentrations of these SCFAs in fecal samples among the groups. The levels of acetic, butyric, isobutyric, and propionic acids were higher in the donor (463.07 ± 29.52, *p* = 0.0088; 53.75 ± 2.72, *p* < 0.0001; 2732.51 ± 160.97, *p* = 0.0011; and 64.31 ± 1.35 mg/ml, *p* = 0.0055, respectively) and FMT_8w (426.05 ± 49.37, *p* = 0.0196; 32.71 ± 0.97, *p* = 0.0013; 1641.24 ± 79.94, *p* = 0.0255; and 53.77 ± 1.86 mg/ml, *p* = 0.0329, respectively) groups than in the before_FMT group (Fig. 3a–d).

### Modulation of the Th1/Th2 balance after FMT

**Fig. 4:**
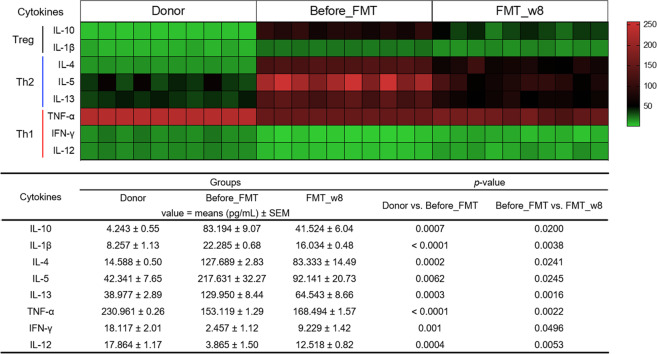
Heatmap showing the effect of FMT treatment on cytokine concentrations. This heatmap presents the individual concentrations of different cytokines, including IL-10, IL-1β, IL-4, IL-5, IL-13, TNF-α, IFN-γ, and IL-12, in the serum, as determined using ELISA kits. The values are depicted using a green to red scale, with green representing lower values and red indicating higher values. ELISA enzyme-linked immunosorbent assay, FMT fecal microbiota transplantation.

Cytokine concentrations were examined in blood serum. The concentrations of Th2 cytokines (IL-4, IL-5, and IL-13), which are known to contribute to the development of AD, were significantly decreased in the FMT_8w group compared with the before_FMT group, whereas the concentrations of Th1 cytokines, such as IL-12, IFN-γ, and TNF-α, were significantly increased (Fig. 4). The levels of cytokines secreted by Tregs (i.e., IL-10 and IL-1β) were significantly lower in the FMT_8w group than in the before_FMT group. These results demonstrate that FMT influences the recovery of the Th1/Th2 balance via Treg signals.

### Improvement of immune modulation after FMT

**Fig. 5:**
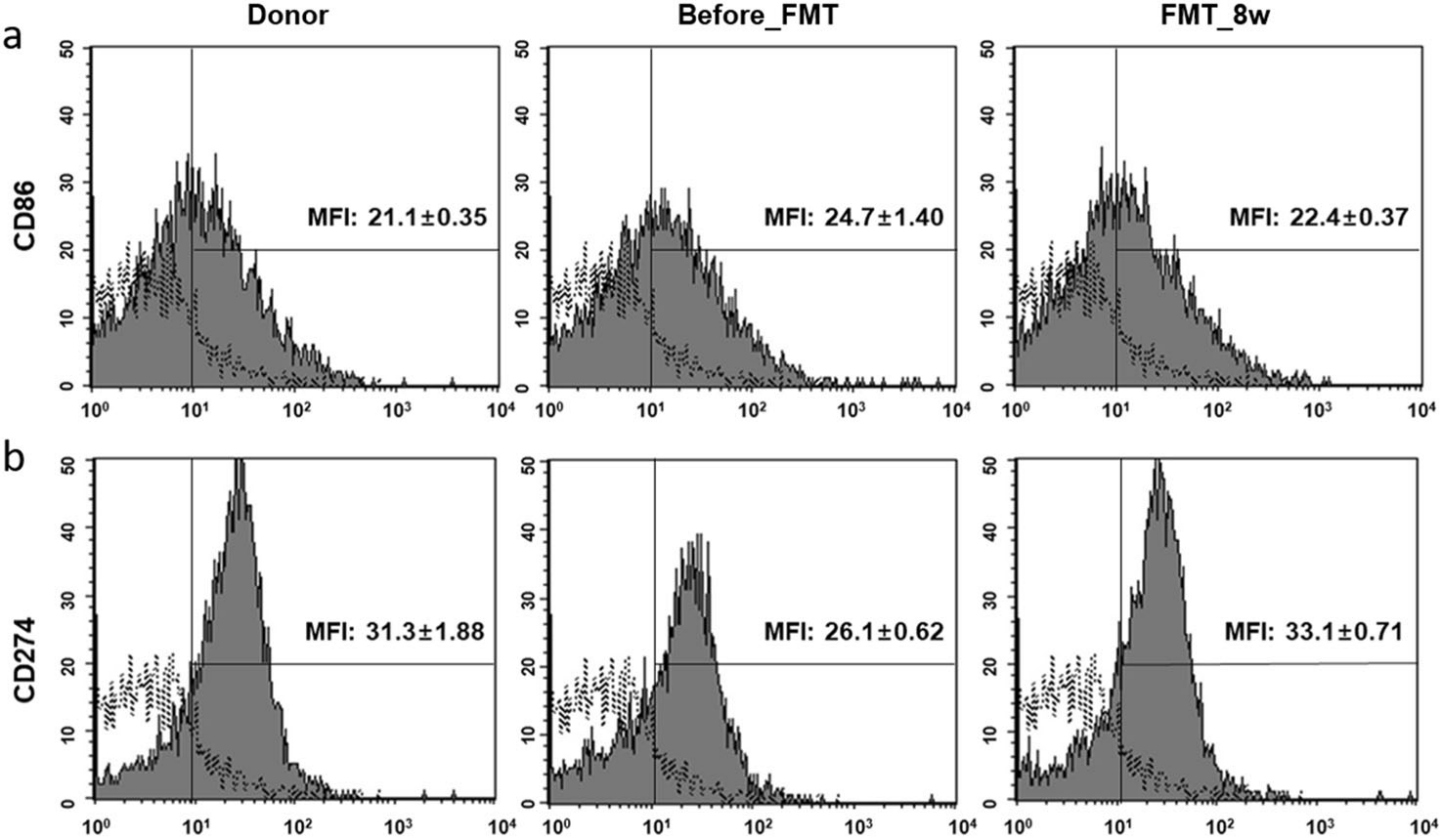
Effect of FMT treatment on T-cell activation, as determined by FACS. T-cell activation was confirmed via flow cytometric analysis. The solid histogram shows isotype antibodies. The filled histogram shows that **a** the MFI of CD 86 was decreased and **b** the MFI of CD 274 was increased in splenocytes in the FMT group compared to the before_FMT group. The unfilled histogram indicates the isotype antibody control. FMT fecal microbiota transplantation, FACS fluorescence-activated cell sorting, MFI mean florescence intensity.

CD 86 and CD 274 expression was evaluated via flow cytometric analysis. CD 86 and CD 274 levels in the FMT and control groups were similar, i.e., CD 86 expression was significantly decreased in the FMT_8w group (22.4 ± 0.37, *p* = 0.0054) compared with the before_FMT group (donor group: 21.1 ± 0.35, *p* = 0.0005; Fig. 5a). However, the level of CD 274 was significantly higher in the FMT_8w group (33.1 ± 0.71, *p* = 0.0003) than in the before_FMT group (donor group: 31.3 ± 1.88, *p* = 0.004) (Fig. 5b).

### Effects of FMT on blood and serum IgE levels

**Fig. 6:**
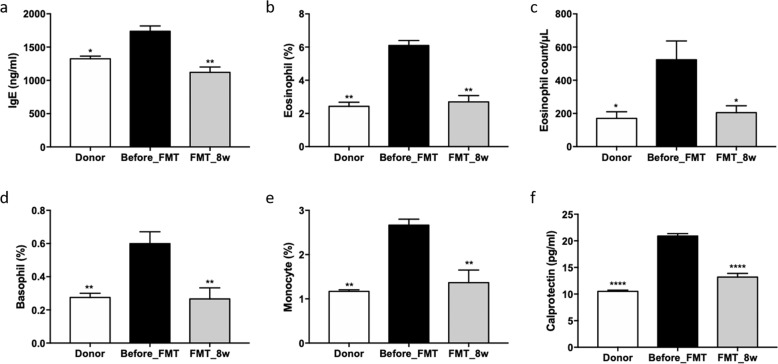
Beneficial effect of FMT on allergy and gut inflammatory factors, as determined by blood tests. **a** The IgE level was examined, and **b** the eosinophil percentage, **c** eosinophil count, **d** basophil percentage, **e** monocyte percentage, and **f** calprotectin level were evaluated via ELISA (*p < 0.05; **p < 0.001; ****p < 0.0001). FMT fecal microbiota transplantation, ELISA enzyme-linked immunosorbent assay.

To confirm the effect of FMT, whole blood and serum IgE analyses were performed. Serum levels of IgE, which is associated with allergic diseases, were significantly decreased in the FMT_8w group (1120.34 ± 66.16 ng/ml, *p* = 0.0025) but increased in the before_FMT group (1737.89 ± 103.84 ng/ml; Fig. 6a). The percentage of eosinophils, eosinophil count, basophil percentage, and monocyte percentage in whole blood were reduced in the FMT_8w group (2.70 ± 0.42%, *p* = 0.0020; 205.00 ± 47.72/μl, *p* = 0.0215; 0.28 ± 0.05%, *p* = 0.0095; and 1.37 ± 0.28%, *p* = 0.0057, respectively) compared with the before_FMT group (6.10 ± 0.17%, 523.33 ± 92.66/μl, 0.60 ± 0.08%, and 2.66 ± 0.13%, respectively; Fig. 6b–e). Moreover, the concentration of calprotectin, a biomarker of inflammation in the gut, was decreased in the FMT_8w group (*p* < 0.0001, 13.19 ± 0.69 pg/ml) compared with the before_FMT group (20.90 ± 0.46 pg/ml; Fig. 6f).

### Reduced inflammation in the skin and gut after FMT

**Fig. 7:**
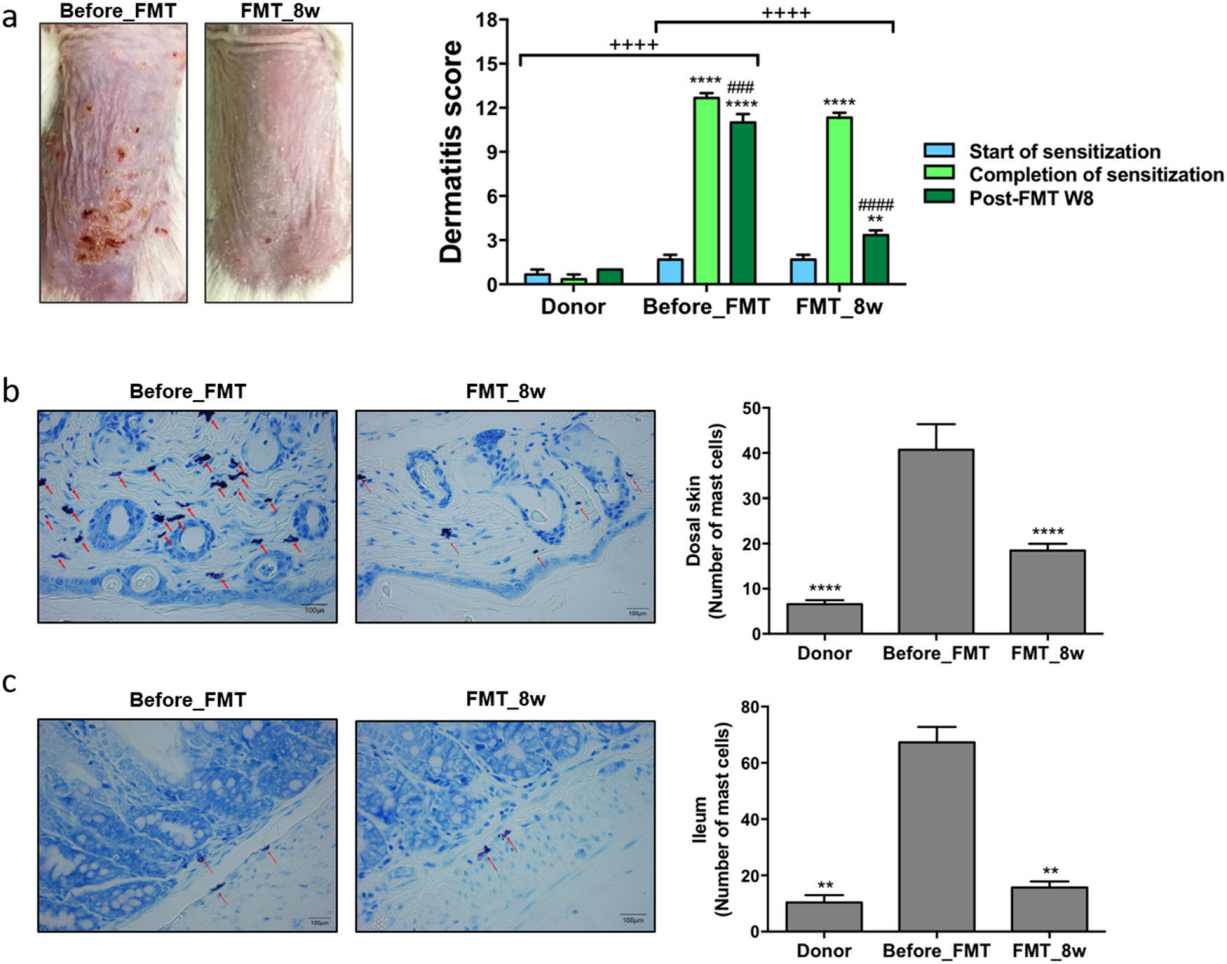
Effect of FMT on inflammation reduction in the skin and gut. **a** AD skin lesions were evaluated, and dermatitis scores were calculating by summing the scores for symptoms (before_FMT group vs FMT_8w group). The number of mast cells in the **b** dorsal skin and **c** ileum was confirmed by toluidine blue staining and quantified microscopically. The red arrows indicate mast cells (**p < 0.001; ^###^*p* < 0.0005; ****, ^####^, and ^++++^*p* < 0.0001). FMT fecal microbiota transplantation, AD atopic dermatitis.

Dermatitis scores were determined and histologic analysis was performed to determine the therapeutic effects of FMT. Significantly lower dermatitis scores were recorded for the FMT_8w group than for the before_FMT group (*p* < 0.0001; Fig. 7a). In addition, lower dermatitis scores were recorded 8 weeks post-FMT than at the time of completion of sensitization (*p* < 0.0001). Infiltrated mast cells in the dorsal skin and ileum lesions were quantified by TB staining, and the results showed that the number of mast cells decreased in the FMT_8w group (*p* < 0.0001 and 0.0018, respectively; Fig. 7b, c). These results indicate that FMT reduced inflammation of skin and gut lesions in mice.

## Discussion

Recent interest in gut microbiota studies has grown, particularly regarding the health benefits of gut microbiota^27^. Many reports have linked gut probiotic bacteria with potential improvements in AD^28^. However, some studies have indicated that probiotics show limited or no significant effects on AD^29,30^, necessitating further studies. FMT is a new and useful approach for altering gut microbiota to effectively treat some disorders, but its effects on allergic diseases, such as AD, remain unknown^14,31^.

In this study, the effects of FMT on gut microbiota restoration were investigated in mice with AD. The FMT_8w group showed an increase in alpha-diversity indices similar to those observed in the donor group. In addition, cluster analysis (unweighted PCoA and a heatmap) showed a similar gut microbiota pattern in the FMT_1w, FMT_8w, and donor groups. FMT led to increased microbial diversity and gut microbiota restoration^32^. A higher abundance of bacterial taxa was identified in the FMT_8w group than in the before_FMT group. Further analysis revealed a high abundance of the families *Porphyromonadaceae*, *Lactobacillaceae*, and *Rikenellaceae* in the donor and FMT_8w groups. An increase in the abundance of these bacterial families may lead to gut microbiota restoration and a shift to a healthy state^33^. These results suggest that gut microbiota restoration via FMT is associated with the amelioration of AD.

To investigate SCFA contents in the gut, fecal pellets from mice were analyzed. SCFAs are associated with the modulation of inflammatory responses^34^. SCFA levels in the FMT_8w group were found to be significantly increased compared with those in the before_FMT group. Acetic acid, butyric acid, and propionic acid are the principal products of carbohydrate fermentation by gut microbiota^35^, and butyric acid, a major metabolite, has been reported to have beneficial effects in AD^36^. These findings are supported by the high abundance of *Lachnospiraceae* and *Ruminococcaceae*^37^. In addition, *Lachnospiraceae*, *Lactobacillaceae*, and *Rikenellaceae* can promote propionate and acetate production^38^. The antiallergic effect of propionic acid in suppressing Th2-mediated allergic airway inflammation was recently reported^39^. Therefore, these results indicate that a high abundance of gut bacteria is associated with an increase in the contents of beneficial metabolites and suggest that FMT has therapeutic effects in AD via gut microbiota restoration and gut health maintenance.

To determine the mechanism underlying the therapeutic effect of FMT on AD, the correlations between gut microbiota abundance, SCFA levels, immune modulation, and the immune response were investigated. The expression of Th2 cytokines (IL-4, IL-5, and IL-13) and Treg cytokines (IL-10 and IL-1β) was downregulated, whereas the expression of Th1 cytokines (IL-12, IFN-γ, and TNF-α) was upregulated, in the FMT_8w compared with the AD group. An increase in butyrate levels was associated with upregulation of Th1 cytokine (IL-12 and IFN-γ) expression and downregulation of Th2 cytokine and IL-10 expression. Propionate and acetate levels are regulated by IFN-γ release^40^. These results connect SCFAs with high-abundance bacterial taxa, such as *Lactobacillus* (the *Lactobacillaceae* family), *Lachnospiraceae*, and *Ruminococcaceae*^41^. The FMT_8w group showed a decrease in calprotectin levels in serum. Although fecal calprotectin was recently shown to be an inflammatory marker in the gut, few AD studies have reported increased calprotectin levels in the skin or serum^42,43^.

IgE is a major indicator of allergic diseases. Mast cells are sensitized by high IgE concentrations and activated to induce an allergic response^44^. In this study, the number of mast cells and the IgE concentration were lower in the FMT_8w group than in the AD group. Low IgE levels contribute to low IL-10 and Th2 cytokine levels and eosinophil, basophil, and monocyte numbers^2,45,46^. The levels of these cytokines were also associated with CD 86 and CD 274 expression. CD 86 expression was significantly downregulated in the FMT_8w group and is regulated in response to IL-10^47^. Conversely, CD 274 expression was upregulated in the FMT_8w group and is linked to IFN-γ^48,49^. Therefore, these results show that FMT is can treat AD by restoring immunologic balance via gut microbiota.

**Fig. 8:**
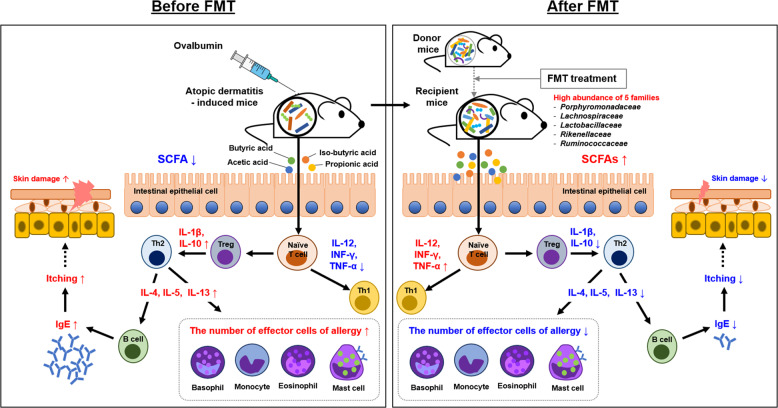
Overview of the mechanism underlying the effect of FMT in atopic dermatitis. The mechanism involved in AD control was proposed by FMT and gut microbiota. Mechanistic links are indicated by solid black lines (—, direct links) and dotted black line (—, speculative link). SCFA short chain fatty acids, AD atopic dermatitis.

In summary, the results of this study indicate that gut microbiota and other parameters, such as cytokine levels, blood parameters, flow cytometry results, histological parameters, and SCFA levels, were significantly restored after FMT treatment (Fig. 8). These findings provide valuable information on the effects of FMT through immune modulation (the Th1/Th2 balance) by gut microbiota, suggesting that FMT may a new therapeutic approach for AD.

## Supplementary information

Supplementary materials

## References

[1] Udkoff J, Waldman A, Ahluwalia J, Borok J, Eichenfield LF (2017). Current and emerging topical therapies for atopic dermatitis. Clin. Dermatol..

[2] Brunner PM, Leung DYM, Guttman-Yassky E (2018). Immunologic, microbial, and epithelial interactions in atopic dermatitis. Ann. Allergy Asthma Immunol..

[3] Wollina U (2017). Microbiome in atopic dermatitis. Clin. Cosmet. Investig. Dermatol..

[4] Craig JM (2016). Atopic dermatitis and the intestinal microbiota in humans and dogs. Vet. Med. Sci..

[5] West CE (2014). Gut microbiota and allergic disease: new findings. Curr. Opin. Clin. Nutr. Metab. Care.

[6] Li D, Wang P, Wang P, Hu X, Chen F (2016). The gut microbiota: a treasure for human health. Biotechnol. Adv..

[7] Cammarota G, Ianiro G, Bibbo S, Gasbarrini A (2014). Gut microbiota modulation: probiotics, antibiotics or fecal microbiota transplantation?. Intern. Emerg. Med..

[8] Cani PD, Delzenne NM (2009). Interplay between obesity and associated metabolic disorders: new insights into the gut microbiota. Curr. Opin. Pharmacol..

[9] Richards JL, Yap YA, McLeod KH, Mackay CR, Marino E (2016). Dietary metabolites and the gut microbiota: an alternative approach to control inflammatory and autoimmune diseases. Clin. Transl. Immunol..

[10] Sampson TR (2016). Gut microbiota regulate motor deficits and neuroinflammation in a model of Parkinson’s disease. Cell.

[11] Rather IA (2016). Probiotics and atopic dermatitis: an overview. Front. Microbiol..

[12] Abrahamsson TR (2012). Low diversity of the gut microbiota in infants with atopic eczema. J. Allergy Clin. Immunol..

[13] Kim H (2015). Oral administration of Lactobacillus plantarum lysates attenuates the development of atopic dermatitis lesions in mouse models. J. Microbiol..

[14] Borody TJ, Khoruts A (2011). Fecal microbiota transplantation and emerging applications. Nat. Rev. Gastroenterol. Hepatol..

[15] Grehan MJ (2010). Durable alteration of the colonic microbiota by the administration of donor fecal flora. J. Clin. Gastroenterol..

[16] Tannock GW (2000). Analysis of the fecal microflora of human subjects consuming a probiotic product containing Lactobacillus rhamnosus DR20. Appl. Environ. Microbiol..

[17] Anderson JL, Edney RJ, Whelan K (2012). Systematic review: faecal microbiota transplantation in the management of inflammatory bowel disease. Aliment. Pharmacol. Ther..

[18] Youngster I (2014). Oral, capsulized, frozen fecal microbiota transplantation for relapsing *Clostridium difficile* infection. JAMA.

[19] Zhou D (2017). Total fecal microbiota transplantation alleviates high-fat diet-induced steatohepatitis in mice via beneficial regulation of gut microbiota. Sci. Rep..

[20] Kim JH, Kim K, Kim W (2019). Cream cheese-derived Lactococcus chungangensis CAU 28 modulates the gut microbiota and alleviates atopic dermatitis in BALB/c mice. Sci. Rep..

[21] Myers-Morales T, Bussell KM, D’Orazio SE (2013). Fecal transplantation does not transfer either susceptibility or resistance to food borne listeriosis in C57BL/6 and BALB/c/By mice. F1000Res.

[22] Li M (2015). Fecal microbiota transplantation and bacterial consortium transplantation have comparable effects on the re-establishment of mucosal barrier function in mice with intestinal dysbiosis. Front. Microbiol..

[23] Bolyen E (2018). QIIME 2: reproducible, interactive, scalable, and extensible microbiome data science. PeerJ Prepr..

[24] Callahan BJ (2016). DADA2: high-resolution sample inference from Illumina amplicon data. Nat. Methods.

[25] Oksanen J (2015). Vegan: community ecology package. R package vegan, vers. 2.2-1. World Agrofor..

[26] McMurdie PJ, Holmes S (2013). phyloseq: an R package for reproducible interactive analysis and graphics of microbiome census data. PLoS ONE.

[27] Mosca A, Leclerc M, Hugot JP (2016). Gut microbiota diversity and human diseases: should we reintroduce key predators in our ecosystem?. Front. Microbiol..

[28] Thomas CL, Fernandez-Penas P (2017). The microbiome and atopic eczema: more than skin deep. Australas. J. Dermatol..

[29] Allen SJ (2014). Probiotics in the prevention of eczema: a randomised controlled trial. Arch. Dis. Child..

[30] Niccoli AA (2014). Preliminary results on clinical effects of probiotic Lactobacillus salivarius LS01 in children affected by atopic dermatitis. J. Clin. Gastroenterol..

[31] Xu MQ (2015). Fecal microbiota transplantation broadening its application beyond intestinal disorders. World J. Gastroenterol..

[32] Cho JA, Chinnapen DJF (2018). Targeting friend and foe: emerging therapeutics in the age of gut microbiome and disease. J. Microbiol..

[33] Rojo D (2017). Exploring the human microbiome from multiple perspectives: factors altering its composition and function. FEMS Microbiol. Rev..

[34] Kespohl M (2017). The microbial metabolite butyrate induces expression of Th1-associated factors in CD4(+) T cells. Front. Immunol..

[35] Liong, M.-T. *Beneficial Microorganisms in Medical and Health Applications*, Vol. 28 (Springer, 2015).

[36] Kim HK (2015). Probiotic supplementation influences faecal short chain fatty acids in infants at high risk for eczema. Benef. Microbes.

[37] Sharma G, Im SH (2018). Probiotics as a potential immunomodulating pharmabiotics in allergic diseases: current status and future prospects. Allergy Asthma Immunol. Res..

[38] Tang C (2019). The impacts of natural polysaccharides on intestinal microbiota and immune responses—a review. Food Funct..

[39] Trompette A (2014). Gut microbiota metabolism of dietary fiber influences allergic airway disease and hematopoiesis. Nat. Med..

[40] Meijer K, de Vos P, Priebe MG (2010). Butyrate and other short-chain fatty acids as modulators of immunity: what relevance for health?. Curr. Opin. Clin. Nutr. Metab. Care.

[41] Million M (2018). New insights in gut microbiota and mucosal immunity of the small intestine. Hum. Microb. J..

[42] Seo SC (2018). Elevated fecal calprotectin levels are associated with severity of atopic dermatitis in children. Asian Pac. J. Allergy Immunol..

[43] Jin S (2014). DAMP molecules S100A9 and S100A8 activated by IL-17A and house-dust mites are increased in atopic dermatitis. Exp. Dermatol..

[44] Liu J (2016). Probiotics enhance the effect of allergy immunotherapy on regulating antigen specific B cell activity in asthma patients. Am. J. Transl. Res..

[45] Gourbeyre P, Denery S, Bodinier M (2011). Probiotics, prebiotics, and synbiotics: impact on the gut immune system and allergic reactions. J. Leukoc. Biol..

[46] Siracusa MC, Kim BS, Spergel JM, Artis D (2013). Basophils and allergic inflammation. J. Allergy Clin. Immunol..

[47] Zhu X (2004). Chronic ethanol ingestion by mice increases expression of CD80 and CD86 by activated macrophages. Alcohol.

[48] Lee (2006). Interferon regulatory factor-1 is prerequisite to the constitutive expression and IFN-gamma-induced upregulation of B7-H1 (CD274). FEBS Lett..

[49] Kim DS (2015). Programmed death-ligand 1, 2 expressions are decreased in the psoriatic epidermis. Arch. Dermatol. Res..

