# Supplementary material for: Gut microbiota restoration through fecal microbiota transplantation: a new atopic dermatitis therapy
Source: Exp Mol Med. 2021 May 20;53(5):907–16. doi: 10.1038/s12276-021-00627-6 (PMC8178377; doi:10.1038/s12276-021-00627-6)
Supplement: Supplementary file 1 — Supplementary materials [file 12276_2021_627_MOESM1_ESM.pdf]

Supplementary materials

Fig. S1. Timeline depicting the various steps in the *in vivo* fecal microbiota transplantation (FMT) experiment. Experimental timeline for FMT from the time of induction of atopic dermatitis using ovalbumin (OVA) sensitization in mice to analysis.

Fig. S2. Taxa level abundance of bacterial taxa in the gut microbiota. The boxplots reveal significant differences in the relative abundance of bacteria (a) phylum, (b) family, and (c) genus among the four experimental groups.

Fig. S3. The heatmap of the top 70 bacterial taxa at genus level based on operational taxonomic units (OTUs). The heatmap reveals increased abundance of *Bacteroidaceae*, *Lactobacillaceae*, *Odoribacteraceae*, and *Rikenellaceae* families in the Donor, FMT_1w, and FMT_8w groups compared with that in the Before_FMT group.

Fig. S1.

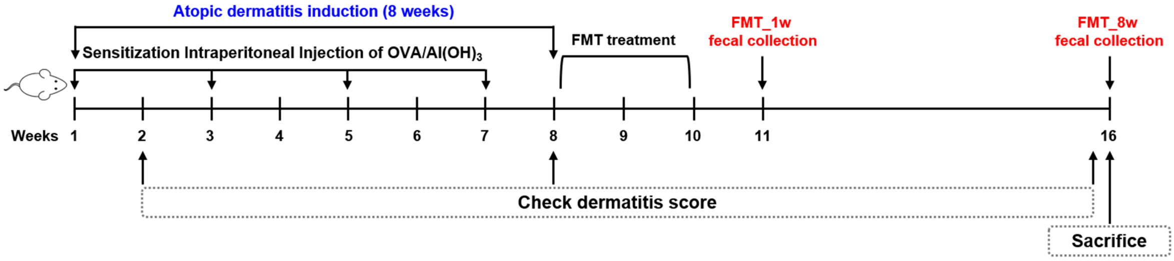

Atopic dermatitis induction (8 weeks)
Sensitization Intraperitoneal Injection of OVA/Al(OH)$_3$
FMT treatment
FMT_1w
fecal collection
FMT_8w
fecal collection
Weeks
1
2
3
4
5
6
7
8
9
10
11
16
Check dermatitis score
Sacrifice

Fig. S2.

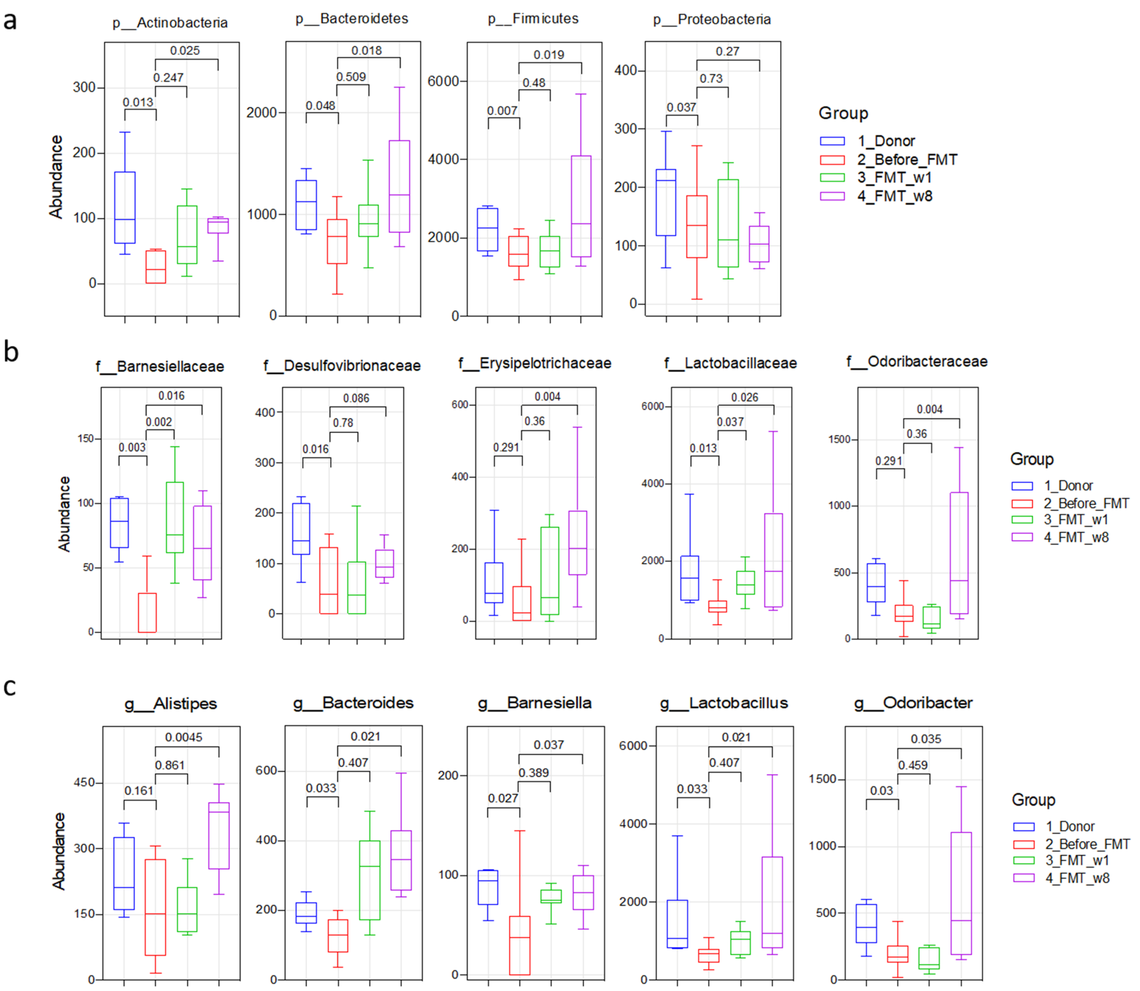
a
p__Actinobacteria
p__Bacteroidetes
p__Firmicutes
p__Proteobacteria
Abundance
0.013
0.247
0.025
0.048
0.509
0.018
0.007
0.48
0.019
0.037
0.73
0.27
Group
1_Donor
2_Before_FMT
3_FMT_w1
4_FMT_w8
b
f__Barnesiellaceae
f__Desulfovibrionaceae
f__Erysipelotrichaceae
f__Lactobacillaceae
f__Odoribacteraceae
Abundance
0.003
0.002
0.016
0.016
0.78
0.086
0.291
0.36
0.004
0.013
0.037
0.026
0.291
0.36
0.004
Group
1_Donor
2_Before_FMT
3_FMT_w1
4_FMT_w8
c
g__Alistipes
g__Bacteroides
g__Barnesiella
g__Lactobacillus
g__Odoribacter
Abundance
0.161
0.861
0.0045
0.033
0.407
0.021
0.027
0.389
0.037
0.033
0.407
0.021
0.03
0.459
0.035
Group
1_Donor
2_Before_FMT
3_FMT_w1
4_FMT_w8

Fig. S3.

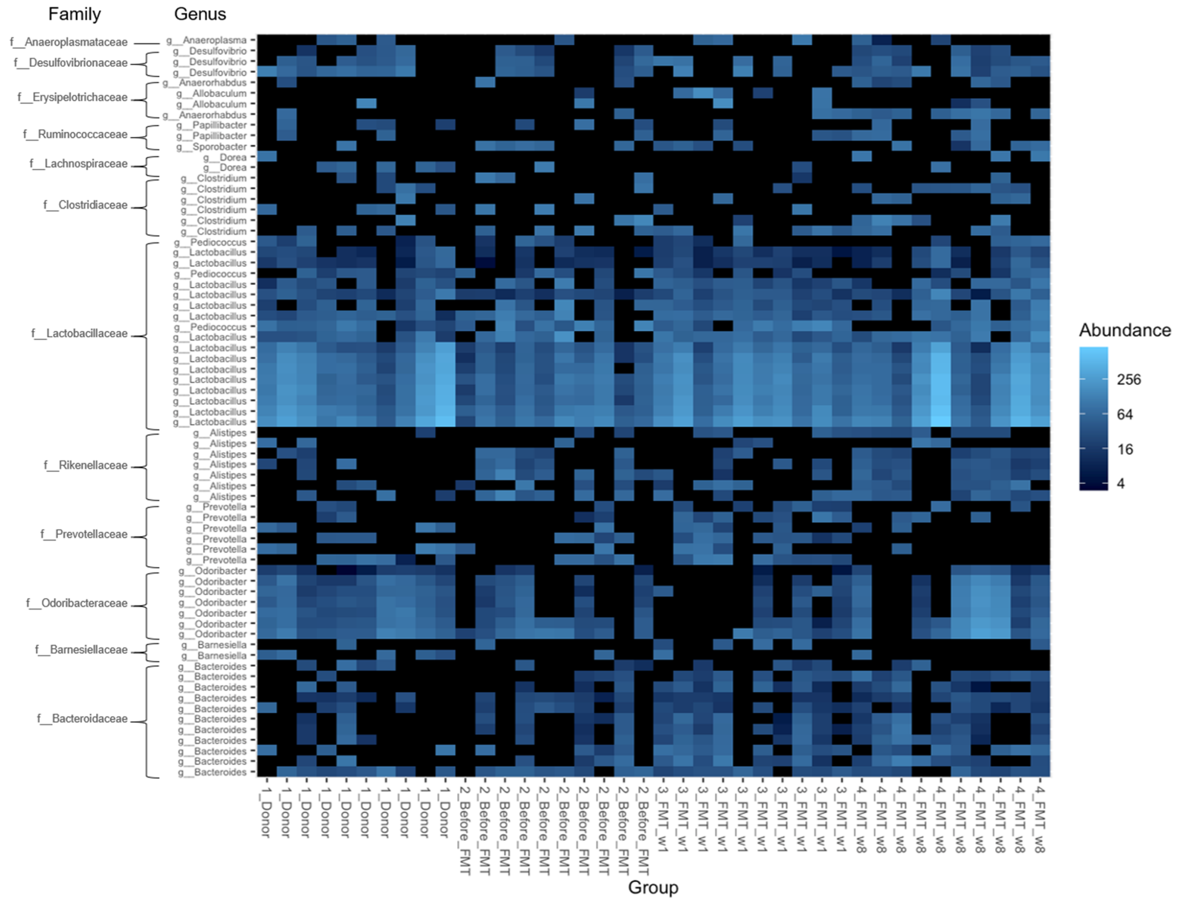
Family
Genus
f__Anaeroplasmataceae
f__Desulfovibrionaceae
f__Erysipelotrichaceae
f__Ruminococcaceae
f__Lachnospiraceae
f__Clostridiaceae
f__Lactobacillaceae
f__Rikenellaceae
f__Prevotellaceae
f__Odoribacteraceae
f__Barnesiellaceae
f__Bacteroidaceae
g__Anaeroplasma
g__Desulfovibrio
g__Anaerorhabdus
g__Allobaculum
g__Papillibacter
g__Sporobacter
g__Dorea
g__Clostridium
g__Pediococcus
g__Lactobacillus
g__Alistipes
g__Prevotella
g__Odoribacter
g__Barnesiella
g__Bacteroides
1_Donor
2_Before_FMT
3_FMT_w1
4_FMT_w8
Group
Abundance
256
64
16
4
